# The Role of Neighborhood Air Pollution Exposure on Somatic Non-Small Cell Lung Cancer Mutations in the Los Angeles Basin (2013–2018)

**DOI:** 10.3390/ijerph191711027

**Published:** 2022-09-03

**Authors:** Noémie Letellier, Sam E. Wing, Jiue-An Yang, Stacy W. Gray, Tarik Benmarhnia, Loretta Erhunmwunsee, Marta M. Jankowska

**Affiliations:** 1 Scripps Institution of Oceanography, University of California San Diego, La Jolla, San Diego, CA 92093, USA; 2 Department of Population Sciences, Beckman Research Institute, City of Hope, Duarte, CA 91010, USA; 3 Department of Population Sciences, City of Hope Comprehensive Cancer Center, Duarte, CA 91010, USA; 4 Department of Surgery, City of Hope Comprehensive Cancer Center, Duarte, CA 91010, USA

**Keywords:** tumor mutations, *TP53* mutation, NSCLC, machine learning, particulate matter

## Abstract

Limited previous work has identified a relationship between exposure to ambient air pollution and aggressive somatic lung tumor mutations. More work is needed to confirm this relationship, especially using spatially resolved air pollution. We aimed to quantify the association between different air pollution metrics and aggressive tumor biology. Among patients treated at City of Hope Comprehensive Cancer Center in Duarte, CA (2013–2018), three non-small cell lung cancer somatic tumor mutations, *TP53*, *KRAS*, and *KRAS G12C*/*V*, were documented. PM_2.5_ exposure was assessed using state-of-the art ensemble models five and ten years before lung cancer diagnosis. We also explored the role of NO_2_ using inverse-distance-weighting approaches. We fitted logistic regression models to estimate odds ratio (OR) and their 95% confidence intervals (CIs). Among 435 participants (median age: 67, female: 51%), an IQR increase in NO_2_ exposure (3.5 μg/m^3^) five years before cancer diagnosis was associated with an increased risk in *TP53* mutation (OR, 95% CI: 1.30, 0.99–1.71). We found an association between highly-exposed participants to PM_2.5_ (>12 μg/m^3^) five and ten years before cancer diagnosis and *TP53* mutation (OR, 95% CI: 1.61, 0.95–2.73; 1.57, 0.93–2.64, respectively). Future studies are needed to confirm this association and better understand how air pollution impacts somatic profiles and the molecular mechanisms through which they operate.

## 1. Introduction

An estimated 131,880 Americans will die from lung cancer in 2021, accounting for 22% of all cancer deaths and making it the leading cause of cancer death in the United States (US) [1]. An important factor associated with lung cancer mortality is tumor biology and the presence of somatic mutations. Some mutations in certain genes can aid in the selection of targeted therapies and lead to improvements in survival outcomes, such as *EGFR* mutations and tyrosine kinase inhibitor treatments [2]. However, mutations in other genes such as *KRAS* and *TP53* are associated with drug resistance [3,4], disease recurrence [5,6], and decreased survival [6,7]. Especially important are *KRAS G12C* and *G12V* mutations, which are associated with a uniquely elevated risk of disease recurrence and decreased overall survival [8,9,10]. While cigarette smoking is a known primary risk factor for *KRAS* and *TP53* mutations [11,12], exposure to environmental pollutants may also be related to the etiology of these mutations [13]. It is important to hone our understanding of the dose–response relationship between environmental pollutants and these deadly lung cancer somatic mutations.

Exposure to ambient air pollution has been linked to both lung cancer risk and mortality, even after accounting for smoking [14,15,16,17]. Fine particulate matter (PM_2.5_) and gaseous pollutants such as nitrogen dioxide (NO_2_) have specifically been implicated in this relationship [18,19,20]. PM_2.5_ is a common urban pollutant, measuring the concentration of ambient particles with an aerodynamic diameter of less than 2.5 µm. PM_2.5_ is a mixture of pollutants originating from a variety of sources, including but not limited to transportation, power generation, and wildfires [21]. NO_2_ is a byproduct of fossil fuel combustion and is frequently used as a proxy for exposure to traffic-related air pollution [22].

Previous research has identified potential biologic processes that can explain the link between ambient air pollution and lung cancer. In vitro work found that exposure to PM_2.5_ at similar concentrations to urban background levels leads to significantly modified cell cycles and altered cell organelles, leading to DNA damage that could ultimately lead to the development of lung cancer [23]. Additionally, PM_2.5_ can induce epigenetic modifications, including DNA methylation linked to the function of bronchial epithelial cells [24]. By contrast, NO_2_ may not be directly carcinogenic [25], but its impact on lung cancer may be due to its high degree of correlation with known traffic-related carcinogens [22,26]. While some previous work has identified a relationship between exposure to ambient air pollution and aggressive somatic lung tumor mutations, more work is needed to confirm this relationship, especially using relevant air pollution assessment.

Measurement of PM_2.5_ relies heavily on the use of the Environmental Protection Agency (EPA) or state-sponsored sensor networks. While highly reliable/accurate and temporally extensive, these networks have course spatial resolution and uneven distribution between urban and rural areas. Models of PM_2.5_ relying solely on course sensor network inputs tend to assume smooth and linear change between the large distances of each node in the network. Yet PM_2.5_ concentrations are subject to hyper-localized variation due to the volatile nature of their sources. Considerable advances have been made in more spatially precise PM_2.5_ models using ensemble models that combine various machine learning algorithms and a large set of predictors including land-use or meteorological data or remote sensing products including aerosol optical depth, for example. It is unclear how a previously estimated dose–response stands when using more refined air pollution models.

In our previous study among patients who were treated at City of Hope Comprehensive Cancer Center (COH) in California (CA), patients living in areas with higher PM_2.5_ exposure had 1.66 (95% CI: 1.02–2.72) increased odds of *TP53*-mutated non-small cell lung cancer (NSCLC) [27]. That study measured air pollution (PM_2.5_ and ozone) with the EPA’s Environmental Justice Screening and Mapping Tool (EJScreen). The EJScreen tool is available at the census tract level and is not available at any period of time, thus limiting the ability to assign air pollution exposures based on the incidence of the disease. It is important to note that in this previous study, PM_2.5_ exposure was assigned in the year or two prior to diagnosis, and neither the role of NO_2_ nor the effect of air pollution on *KRAS* mutations were investigated.

In this present study, we aim to improve on previous research by relying on spatially- and temporally-resolved PM_2.5_ exposure. We used a state-of-the-art ensemble model for PM_2.5_ that we recently developed for California (combining multiple machine learning algorithms). We also explored the role of NO_2_ using traditional approaches based on inverse-distance-weighting. We assessed PM_2.5_ and NO_2_ exposures 5 and 10 years prior to cancer diagnosis.

## 2. Materials and Methods

### 2.1. Study Design and Participants

We reviewed all patients with a primary NSCLC diagnosis who were treated at City of Hope Comprehensive Cancer Center (COH) in Duarte, CA, USA, from 2013 through to 2018. We included patients in this analysis if they had received somatic *TP53* or *KRAS* sequencing documented in the electronic medical record (EMR) and had a valid home address. Patients with non-US addresses or PO boxes were not included. We excluded patients with (i) diagnosis of small cell lung cancer, carcinoid tumors, or sarcomas; (ii) in situ lung cancer; (iii) <18 years of age; and/or (iv) multiple primary NSCLCs with different somatic phenotypes.

Patients included in the study provided written consent, and the study was approved by the COH Institutional Review Board and conducted in accordance with the International Ethical Guidelines for Biomedical Research Involving Human Subjects.

### 2.2. Air Pollution Exposure Assessment

Data on concentrations of fine particulate matter < 2.5 µm (PM_2.5_) and nitrogen dioxide (NO_2_) in µg/m^3^ were routinely collected by US Environmental Protection Agency through ambient air pollutant monitoring stations. For PM_2.5_ exposure, we relied on an ensemble model we recently developed for California [28]. Briefly, we estimated daily levels of PM_2.5_ at the ZIP code level using a validated ensemble model combining multiple machine learning algorithms (e.g., random forest, gradient boosting) and multiple predictors (e.g., meteorological factors such as temperature, precipitation or wind patterns, satellite-derived aerosol optical depth, or land-use variables). For NO_2_, we relied on a traditional inverse-distance-weighting (IDW) method to estimate daily NO_2_ concentrations at the ZIP code population-weighted centroid. We used PM_2.5_ and NO_2_ annual concentrations five and ten years before lung cancer diagnosis based on participants’ home addresses.

### 2.3. Covariates

Data on patient demographics and clinical characteristics were obtained from the COH hospital-based cancer registry. Sociodemographic characteristics included age (continuous), sex (female or male), race/ethnicity (Asian, Black, Hispanic, or Non-Hispanic White), educational attainment (<HS grad, HS grad, college degree, or graduate degree), insurance status (Medicaid or not Medicaid), and cigarette smoking (current, former, or never). Clinical characteristics included cancer stage (I, II, III, or IV), cancer histology (adenocarcinoma, squamous, or other) and year of lung cancer diagnosis (from 2013 to 2018). We also assigned patients an estimated exposure to neighborhood-level socioeconomic status based on their home address using the Area Deprivation Index. Briefly, this measure ranks a census block group’s disadvantage within a given state, as measured by a composite of the area’s income, education, employment, and housing quality [29].

### 2.4. Outcomes

The main outcomes of interest are the following 3 NSCLC somatic tumor mutations: *TP53*, all *KRAS* mutations, and *KRAS G12C* and *G12V* mutations (*KRAS G12C/V*). Somatic genomic tests were ordered as part of usual clinical care and sequencing results were obtained from the COH EMR, which contains test results from both internal and external laboratories. Results were typically generated from either the COH Clinical Molecular Diagnostics Laboratory, Foundation Medicine, Inc. (San Diego, CA, USA), or Guardant Health, Inc. (Redwood City, CA, USA). For patients who received multiple tests but had discrepant results, study staff prioritized findings from tissue over blood-based assays.

### 2.5. Statistical Analysis

To assess association between air pollution and lung cancer tumor mutations, we fit logistic regression models to estimate odds ratio (OR) and their 95% confidence intervals (CIs) per interquartile range (IQR) increase in PM_2.5_ and NO_2_ concentrations. Separate models were considered for each combination of air pollution estimates and tumor mutations for five and ten years prior to diagnosis. First, the crude association between air pollution exposure and cancer tumor mutations was investigated. Then, all models were adjusted for previously identified cofounders: age (in continuous), sex, race/ethnicity, educational level, insurance status, area deprivation level, smoking status, cancer stage, cancer histology, and year of diagnosis.

In supplementary analyses, we categorized the air pollution estimates (PM_2.5_ and NO_2_) in tertiles. Then, we used absolute cutoffs to define high exposure to PM_2.5_ according to the US EPA guidelines [30]. When the annual PM_2.5_ concentration was higher than 12 μg/m^3^, participants were classified as high exposed.

Missing data were observed on a few of the variables we assessed (Table S1). In sensitivity analyses, missing data for exposures and covariates were handled using multiple imputations by chained equations (MICE) package in R with 10 imputed datasets [31].

All analyses were performed using R, version 3.6.0.

## 3. Results

### 3.1. Characteristics of the Study Sample

Among the 694 participants included at baseline, we restricted the population to participants with *TP53* or *KRAS* data and with air pollution exposure data (PM_2.5_ or NO_2_). The sample selection is explained in Figure S1. The characteristics of the 435 participants included in this study are described in Table 1. The average age was 67 years (SD, 12), 51% were female and 42% had at least some college education. The majority of participants were non-Hispanic White (56%), followed by Asian participants (31%), Hispanic White (8.5%), and Black (4.6%). Few participants in the study population (7%) reported receiving Medicaid insurance. The year of lung cancer diagnosis was from 2013 to 2018. Sixteen percent of the participants were current smokers, 46% were former smokers, and 38% had never smoked. Most participants had stage IV lung cancer (70%) and a lung adenocarcinoma diagnosis (86%).

### 3.2. Air Pollution Level 5 and 10 Y Prior to Diagnosis

The average PM_2.5_ level for all participants was 13.9 μg/m^3^ (IQR, 12.2–15.6) five years before lung cancer diagnosis, and 13.9 μg/m^3^ (IQR, 12.2–15.5) ten years before lung cancer diagnosis. The average NO_2_ level was 17 μg/m^3^ (IQR, 14.8–18.3) five years before diagnosis, and 18.9 μg/m^3^ (IQR, 16.3–20.5) ten years before diagnosis.

The average air pollution level according to each mutation status is shown in Table 2. Among 409 participants with complete data for *TP53* mutation and PM_2.5_ exposure, 238 were positive for the *TP53* mutation with an average PM_2.5_ level five years prior to diagnosis at 14.1 μg/m^3^ (IQR, 12.7–15.7). The average PM_2.5_ level five years prior to diagnosis was at 13.9 μg/m^3^ (IQR, 11.8–15.6) for participants without *TP53* mutation (*n* = 171).

### 3.3. Association between Air Pollution and Lung Cancer Tumor Mutations

Table 3 shows crude and adjusted odds ratios and 95% confidence intervals for *TP53*, *KRAS*, and *KRAS G12C/V* mutation status for every IQR (3.3 μg/m^3^) increase in PM_2.5_ exposure five or ten years prior to cancer diagnosis. In crude and adjusted models, an IQR increase in PM_2.5_ exposure five or ten years before diagnosis was not significantly associated with overall mutation status. However, the odds ratio was higher for *TP53* mutation status with adjusted ORs (95% CI) of 1.24 (0.93–1.67) for five years prior to diagnosis and of 1.25 (0.93–1.67) for ten years prior to diagnosis.

Crude and adjusted ORs and 95% confidence intervals for overall mutation status and NO_2_ exposure five and ten years prior to cancer diagnosis for an IQR increase (3.5 μg/m^3^ for five years and 4.2 μg/m^3^ for ten years prior to diagnosis) are shown in Table 3. An increase of 3.5 μg/m^3^ in NO_2_ exposure five years before cancer diagnosis was associated with *TP53* tumor mutation (OR: 1.30, 95% CI: 0.99, 1.71). No associations were highlighted for the two *KRAS* mutations five and ten years before cancer diagnosis.

**Figure 1:**
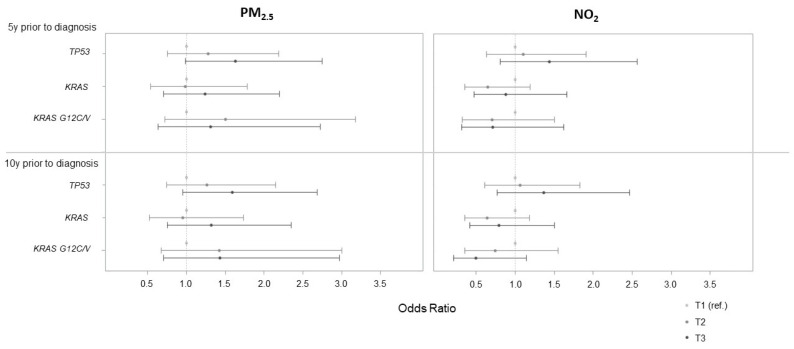
Associations between PM_2.5_ and NO_2_ categorized in tertile (assessed 5 and 10 years prior to cancer diagnosis) and lung cancer tumor mutations. Based on tertile distribution, PM_2.5_ 5 y prior to diagnosis was classified as [6.4;13.1], (13.1;15.0], and (15.0;19.6]; PM_2.5_ 10 y prior to diagnosis as [6.3;13.0], (13.0;15.0], and (15.0;19.6]; NO_2_ 5 y prior to diagnosis as [2.9;16.1], (16.1;17.8], and (17.8;21.5]; and NO_2_ 10 y prior to diagnosis as [3.4;17.5], (17.5;19.9], and (19.9;24.6].

Using the lowest tertile as the reference, the adjusted OR (95% CI) evaluating the association between PM_2.5_ exposure five years before diagnosis and *TP53* tumor mutation was 1.63 (0.98, 2.75) for the highest tertile, corresponding to an exposure higher than 15 μg/m^3^ (Figure 1). No association was observed with the other mutation status and for NO_2_ exposure in tertile.

**Figure 2:**
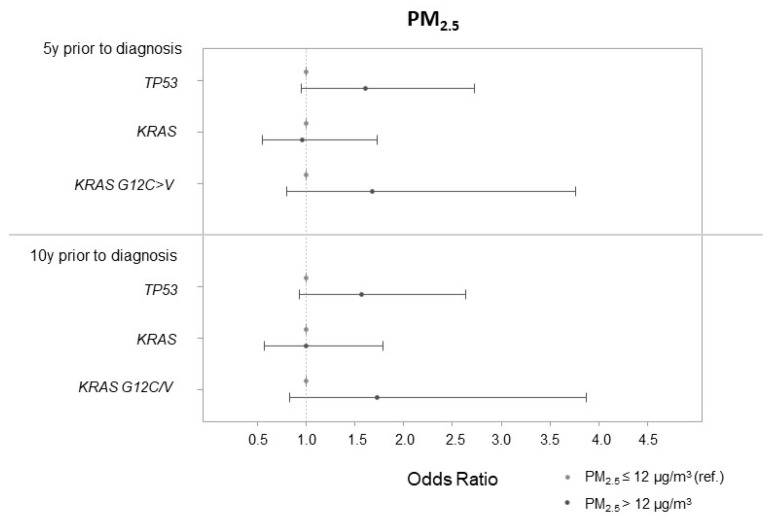
Associations between PM_2.5_ in binary according to US EPA guidelines (PM_2.5_ > 12 μg/m^3^ 5 and 10 years prior to cancer diagnosis) and lung cancer tumor mutations.

For the association between highly exposed participants to PM_2.5_ (i.e., with an exposure higher than 12 μg/m^3^, US EPA guidelines) five and ten years before cancer diagnosis and TP53 mutation, the adjusted ORs (95% CI) were 1.61 (0.95, 2.73) and 1.57 (0.93, 2.64), respectively (Figure 2).

When we performed multiple imputation to handle missing data for exposures and covariates, the ORs were weaker and more imprecise (Table S2).

## 4. Discussion

In this study, we investigated the associations between air pollution concentration level and somatic non-small cell lung cancer mutations: *TP53*, *KRAS*, and *KRAS G12C/V* mutations. Among approximately 400 participants, the associations appeared to be higher between air pollution level (PM_2.5_ and NO_2_ exposure) five years before cancer diagnosis and *TP53*-mutated NSCLC. In contrast, no association was found between air pollution estimates and *KRAS* and *KRAS G12C/V* mutations.

The association between air pollution and lung cancer incidence is well established [14,15,16,17]. Due to sufficient evidence for a causal association between particulate matter and an increased risk of lung cancer, air pollution was classified as a carcinogen by the World Health Organization International Agency for Research on Cancer (IARC) in 2013 [32]. The mixture of carcinogenic and mutagenic substances present in PM, such as benzo(a)pyrene (BaP) and polycyclic aromatic hydrocarbons (PAHs), can be metabolized in the body and cause DNA damage, genomic instability, and promote malignant neoplasms [30,33]. The NSCLC mutations are induced by DNA adducts that are formed by the release of reactive intermediates when BaP and other PAHs are metabolized. Inhalation of PM_2.5_ particles may attract lymphocytes to tissues, resulting in angiogenesis and inflammation that could promote tumor growth [34,35].

Limited previous work has identified a relationship between exposure to ambient air pollution and aggressive somatic lung tumor mutations. In our study, we found that environmental pollutants (PM_2.5_ and NO_2_) five years before cancer diagnosis could be related to the etiology of the *TP53* mutation. However, we did not find any association with other NSCLC somatic tumor mutations (*KRAS* and *KRAS G12C/V)*, thus requiring further investigations. The biological mechanism of if and how exposure to air pollution impacts NSCLC biology is not clear. An association between air pollution and *TP53* mutations has been observed in both mouse models and human cell lines [36,37,38]. *TP53* mutations have been observed in mouse cell lines that were experimentally exposed to different environmental toxins, such as BaP and 3-nitrobenzanthrone [37]. In a previous in vitro study, human cell lines exposed to 3-nitrobenzanthrone (a component of diesel exhaust) presented numerous mutations in *TP53* [39].

To the best of our knowledge, only two epidemiological studies have studied the link between outdoor air pollution and specific NSCLC mutations. In a cohort of patients living in China, an association between highly-polluted regions and specific somatic NSCLC mutations was reported [40]. Patients who lived in highly-polluted regions had three times higher mutated genes, including *TP53*, as those in control, lower-pollution regions. Our previous work found that *TP53*-mutated NSCLC was linked to areas with higher PM_2.5_ exposure [27]. However, this work only focused on *TP53*-mutated NSCLC and on two pollutants (PM_2.5_ and ozone), which were assessed through EPA’s EJScreen in the year or two prior to cancer diagnosis. We hope to now expand that work by including two other NSCLC somatic tumor mutations (*KRAS* and *KRAS G12C/V),* and by overcoming several limitations of previous studies using more a precise exposure assessment and by limiting the exposure misclassification. We used improved estimation methods to assess exposure to PM2.5 [28]. Moreover, because health effects can be caused by both short-term exposure and long-term exposure to pollutants, we looked at PM_2.5_ and NO_2_ concentration levels up to ten years prior to cancer diagnosis.

Our study has some limitations. First, due to the relatively small number of participants (~400), we are insufficiently powered to conduct analyses stratified by stage of diagnosis or cancer histology (adenocarcinoma, squamous, other). Moreover, we focused on a small number of genes, *TP53* and *KRAS*. We do not have the historic residential patients’ addresses prior to diagnosis, so we were unable to assess patients’ previous exposure to carcinogens. We do not consider potential co-exposure, other than smoking, while other environmental risk factors, such as exposure to radon, household pollutants, and occupational exposure, could be important to take into account as a person may be exposed to several and often synergistic exposures [41]. Finally, in our study a third of the patients were non-smoking Asians and only a few Hispanic Americans and African Americans were included. This may impact the generalizability of these findings to other populations with different racial/ethnic compositions. Our study also has some strengths including the study sample with almost 400 participants with tumor sequencing results and smoking data, which is a relatively large sample that contains information on somatic oncogenic molecular abnormalities [41]. Moreover, we adjusted for potential cofounders that have previously been omitted, including smoking status and area deprivation level.

## 5. Conclusions

Even if the associations were at the limit of significance, our results suggest that the concentration of environmental pollutants (PM_2.5_ and NO_2_) five years before cancer diagnosis is associated with *TP53*-mutated NSCLC, using improved estimation methods to assess air pollution. In contrast, we did not find any association with *KRAS* and *KRAS G12C/V* mutations. Future studies are needed to confirm this association and better understand how air pollution affects somatic profiles and through which molecular mechanisms. This improved understanding could help better identify individuals who may be at high risk of developing aggressive disease, implement accurate screening of high-risk patients, and improve the use of targeted therapies.

## Figures and Tables

**Table 1:** Characteristics distribution of selected participants from the CCPS data (*n* = 435).

Characteristic, *n* (%)	
Age, mean (SD)	67.2 (12.0)
Female	224 (51)
Race/ethnicity	
Asian	133 (31)
Black	20 (4.6)
Hispanic White	37 (8.5)
Non-Hispanic White	245 (56)
Educational attainment	
<HS grad	45 (10)
HS grad	206 (47)
College degree	118 (27)
Grad degree	66 (15)
Insurance	
Medicaid	29 (6.7)
Not Medicaid	406 (93)
Area deprivation level, median (IQR)	4 (2–6)
Smoking status	
Current smoker	68 (16)
Former smoker	200 (46)
Never smoker	167 (38)
Cancer stage	
I–IIB	63 (14)
IIIA–IIIB	69 (16)
IV	303 (70)
Cancer histology	
Adenocarcinoma	373 (86)
Squamous cell carcinoma	33 (7.6)
Other	29 (6.7)

**Table 2:** Distribution of PM_2.5_ and NO_2_ exposure according to mutation status.

Median (IQR)		PM_2.5_ Exposure			NO_2_ Exposure	
	*n*	5 Years before Diagnosis	10 Years before Diagnosis	*n*	5 Years before Diagnosis	10 Years before Diagnosis
TP53 mutation status	409			380		
Negative	171	13.9 (11.8, 15.6)	13.9 (11.8, 15.5)	158	16.9 (14.6, 18.6)	18.9 (16.4, 20.9)
Positive	238	14.1 (12.7, 15.7)	14.1 (12.6, 15.6)	222	17.2 (15.0, 18.3)	19.4 (16.9, 20.6)
KRAS mutation status	435			405		
Negative	313	13.9 (12.5, 15.6)	13.9 (12.4, 15.5)	291	17.0 (15.2, 18.2)	19.0 (16.9, 20.5)
Positive	122	14.3 (12.6, 15.7)	14.4 (12.5, 15.6)	114	17.1 (14.3, 18.6)	19.0 (15.9, 21.1)
KRAS G12C/V status	435			405		
Negative	370	13.9 (12.5, 15.6)	13.9 (12.3, 15.5)	344	17.1 (15.1, 18.3)	19.1 (16.7, 20.6)
Positive	65	14.3 (12.9, 15.6)	14.3 (12.8, 15.5)	61	16.9 (14.3, 18.1)	18.7 (15.6, 20.3)

**Table 3:** Association between PM_2.5_ and NO_2_ concentrations 5 and 10 years before cancer diagnosis and lung cancer tumor mutations.

	Crude	Adjusted
	OR (CI 95%)	OR (CI 95%)
PM_2.5_ exposure		
TP53 mutation status (*n* = 409)		
PM_2.5_ exposure 5 years before diagnosis	1.19 (0.91–1.55)	1.24 (0.93–1.67)
PM_2.5_ exposure 10 years before diagnosis	1.19 (0.91–1.56)	1.25 (0.93–1.67)
KRAS mutation status (*n* = 435)		
PM_2.5_ exposure 5 years before diagnosis	1.08 (0.81–1.45)	1.13 (0.82–1.57)
PM_2.5_ exposure 10 years before diagnosis	1.08 (0.81–1.45)	1.13 (0.82–1.57)
KRAS G12C/V mutation status (*n* = 435)		
PM_2.5_ exposure 5 years before diagnosis	1.08 (0.75–1.57)	1.20 (0.81–1.80)
PM_2.5_ exposure 10 years before diagnosis	1.08 (0.76–1.58)	1.21 (0.82–1.82)
NO_2_ exposure		
TP53 mutation status (*n* = 380)		
NO_2_ exposure 5 years before diagnosis	1.24 (0.97–1.59)	1.30 (0.99–1.71)
NO_2_ exposure 10 years before diagnosis	1.23 (0.95–1.60)	1.30 (0.97–1.76)
KRAS mutation status (*n* = 405)		
NO_2_ exposure 5 years before diagnosis	0.96 (0.74–1.25)	0.93 (0.69–1.26)
NO_2_ exposure 10 years before diagnosis	0.99 (0.75–1.32)	0.96 (0.69–1.33)
KRAS G12C/V mutation status (*n* = 405)		
NO_2_ exposure 5 years before diagnosis	0.90 (0.66–1.26)	0.95 (0.65–1.42)
NO_2_ exposure 10 years before diagnosis	0.89 (0.64–1.27)	0.94 (0.62–1.44)

PM_2.5_ exposure assessed by machine learning estimates and NO_2_ exposure assessed by IDW method. Models adjusted for age, sex, race/ethnicity, educational level, insurance status, area deprivation index, smoking status, cancer stage, cancer histology, and year of diagnosis.

## Data Availability

The datasets used for analyses during the current study are not publicly available due to ethical restrictions and participant confidentiality but are available from the corresponding author on reasonable request.

## Supplementary Materials

The following supporting information can be downloaded at: https://www.mdpi.com/article/10.3390/ijerph191711027/s1, Figure S1. Sample selection; Table S1. Distribution of missing data; Table S2. Association between PM_2.5_ and NO_2_ concentrations 5 and 10 years before cancer diagnosis and lung cancer tumor mutations after multiple imputation.
