# Supplementary material for: The Role of Neighborhood Air Pollution Exposure on Somatic Non-Small Cell Lung Cancer Mutations in the Los Angeles Basin (2013–2018)
Source: Int J Environ Res Public Health. 2022 Sep 3;19(17):11027. doi: 10.3390/ijerph191711027 (PMC9518136; doi:10.3390/ijerph191711027)
Supplement: Supplementary file 1 [file ijerph-19-11027-s001.zip › ijerph-1850557-supplementary.pdf]

Supplementary data

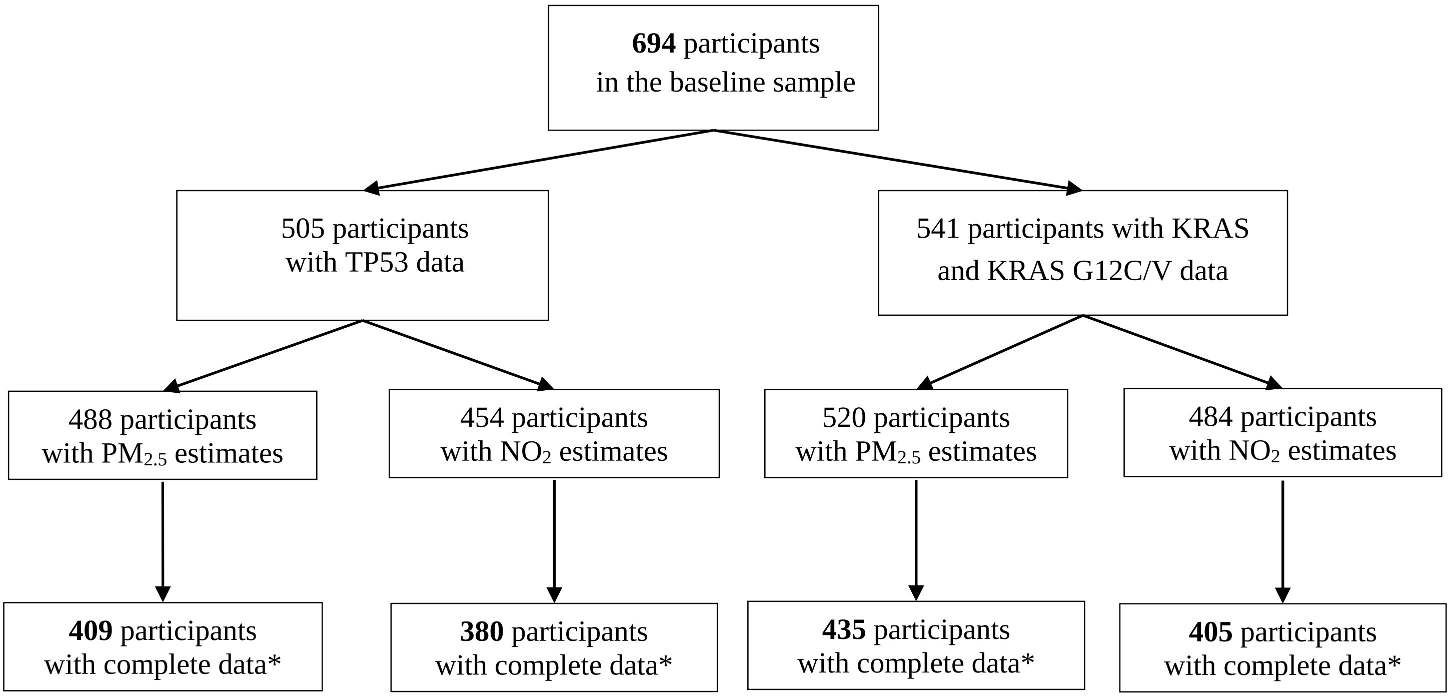

Figure S1. Sample selection. *Complete data for covariates (age, sex, race/ethnicity, educational level, insurance status, area deprivation level, smoking status, cancer stage, cancer histology and year of diagnosis).

Table S1. Distribution of missing data.

Number of missing data, n (%)	Participants with TP53 data (N = 505)	Participants with KRAS data (N = 541)
Exposures		
IDW method		
NO_2 five years before diagnosis	51 (10.1)	57 (10.5)
NO_2 ten years before diagnosis	50 (9.9)	56 (10.4)
ML estimates		
$PM_{2.5}$ five years before diagnosis	17 (3.4)	21 (3.4)
$PM_{2.5}$ ten years before diagnosis	17 (3.4)	21 (3.4)
Covariates		
Age	0 (0.0)	0 (0.0)
Sex	0 (0.0)	0 (0.0)
Race/ethnicity	11 (2.2)	13 (2.4)
Educational attainment	62 (12.3)	69 (12.8)
Insurance	2 (0.4)	2 (0.4)
Smoking status	0 (0.0)	0 (0.0)
Cancer stage	7 (1.4)	6 (1.1)
Cancer histology	0 (0.0)	0 (0.0)
Year of diagnosis	0 (0.0)	0 (0.0)
Area deprivation index	16 (3.2)	21 (3.9)

Table S2. Association between $PM_{2.5}$ and NO_2 concentrations 5 and 10 years before cancer diagnosis and lung cancer tumor mutations after multiple imputation

	**Crude** OR (CI 95%)	**Adjusted**[b] OR (CI 95%)
$PM_{2.5}$ exposure[a]		
TP53 mutation status (N=505)		
$PM_{2.5}$ exposure 5 years before diagnosis	1.08 (0.84 – 1.37)	1.12 (0.85 – 1.46)
$PM_{2.5}$ exposure 10 years before diagnosis	1.10 (0.86 – 1.40)	1.14 (0.88 – 1.49)
KRAS mutation status (N=541)		
$PM_{2.5}$ exposure 5 years before diagnosis	0.97 (0.75 – 1.26)	1.06 (0.79 – 1.42)
$PM_{2.5}$ exposure 10 years before diagnosis	1.00 (0.77 – 1.30)	1.06 (0.79 – 1.43)
KRAS G12C/V mutation status (N=541)		
$PM_{2.5}$ exposure 5 years before diagnosis	0.95 (0.69 – 1.32)	1.09 (0.76 – 1.54)
$PM_{2.5}$ exposure 10 years before diagnosis	0.97 (0.70 – 1.34)	1.09 (0.76 – 1.56)
NO_2 exposure[a]		
TP53 mutation status (N=505)		
$PM_{2.5}$ exposure 5 years before diagnosis	1.15 (0.93 – 1.42)	1.18 (0.94 – 1.48)
$PM_{2.5}$ exposure 10 years before diagnosis	1.12 (0.90 – 1.41)	1.16 (0.91 – 1.49)
KRAS mutation status (N=541)		
$PM_{2.5}$ exposure 5 years before diagnosis	0.96 (0.77 – 1.20)	1.01 (0.78 – 1.31)
$PM_{2.5}$ exposure 10 years before diagnosis	0.98 (0.77 – 1.25)	1.03 (0.78 – 1.37)
KRAS G12C/V mutation status (N=541)		
$PM_{2.5}$ exposure 5 years before diagnosis	0.94 (0.71 – 1.23)	1.01 (0.73 – 1.38)
$PM_{2.5}$ exposure 10 years before diagnosis	0.92 (0.69 – 1.23)	0.98 (0.69 – 1.38)

[a]$PM_{2.5}$ exposure assessed by machine learning estimates and NO_2 exposure assessed by IDW method
[b]Models adjusted for age, sex, race/ethnicity, educational level, insurance status, area deprivation index, smoking status, cancer stage, cancer histology and year of diagnosis
